# Global and regional incidence of intrahepatic cholestasis of pregnancy: a systematic review and meta-analysis

**DOI:** 10.1186/s12916-025-03935-0

**Published:** 2025-02-28

**Authors:** Ali Jamshidi Kerachi, Mohammad Amin Shahlaee, Pardis Habibi, Niloofar Dehdari Ebrahimi, Moein Ala, Alireza Sadeghi

**Affiliations:** 1 Student Research Committee, Shiraz University of Medical Sciences, Shiraz, Iran; 2 Transplant Research Center, Shiraz University of Medical Sciences, Shiraz, Iran; 3 Experimental Medicine Research Center, School of Medicine, Tehran University of Medical Sciences, Tehran, Iran; 4 Gastroenterohepatology Research Center, Shiraz University of Medical Sciences, Shiraz, Iran

**Keywords:** Incidence, Systematic review, Meta-analysis, Intrahepatic cholestasis of pregnancy

## Abstract

**Background:**

Intrahepatic cholestasis of pregnancy (ICP) can be a source of significant distress for both pregnant women and the fetus, impairing the quality of life and well-being of pregnant women, leading to psychological disorders among pregnant women with severe or recurrent ICP, and causing life-threatening complications among fetuses. Regrettably, our current understanding of ICP globally is limited, lacking a comprehensive estimation of its incidence. Therefore, in this systematic review and meta-analysis, we aimed to investigate the global and regional incidence of ICP and identify factors that account for its variety across studies.

**Methods:**

A comprehensive search strategy was implemented across PubMed, Scopus, and Web of Science databases. To stabilize the variance, the Freeman-Tukey double arcsine transformation was employed. Subgroup analyses were conducted based on continent, publication type, study design and timing, regional classifications, developmental status, and World Bank income grouping. A multivariate meta-regression analysis was performed to estimate the effects of the continuous moderators on the effect size.

**Results:**

A total of 42,972,872 pregnant women were analyzed from 302 studies. The overall pooled incidence [95% confidence interval] of ICP was 2.9% [2.5, 3.3]. Studies with larger sample sizes tended to provide significantly lower estimates of ICP incidence: 1.6% [1.3, 2] vs 4.7% [3.9, 5.5]. Asia had the highest incidence of ICP among the continents, whereas Oceania had the lowest. Countries that were classified as developed and with higher income had a lower incidence of ICP than those classified as developing and low and middle income.

**Conclusions:**

The findings of this study will provide valuable insights into the current knowledge regarding the association of the quality of public health and socioeconomic variations with the incidence of ICP on a global scale.

**Graphical Abstract:**

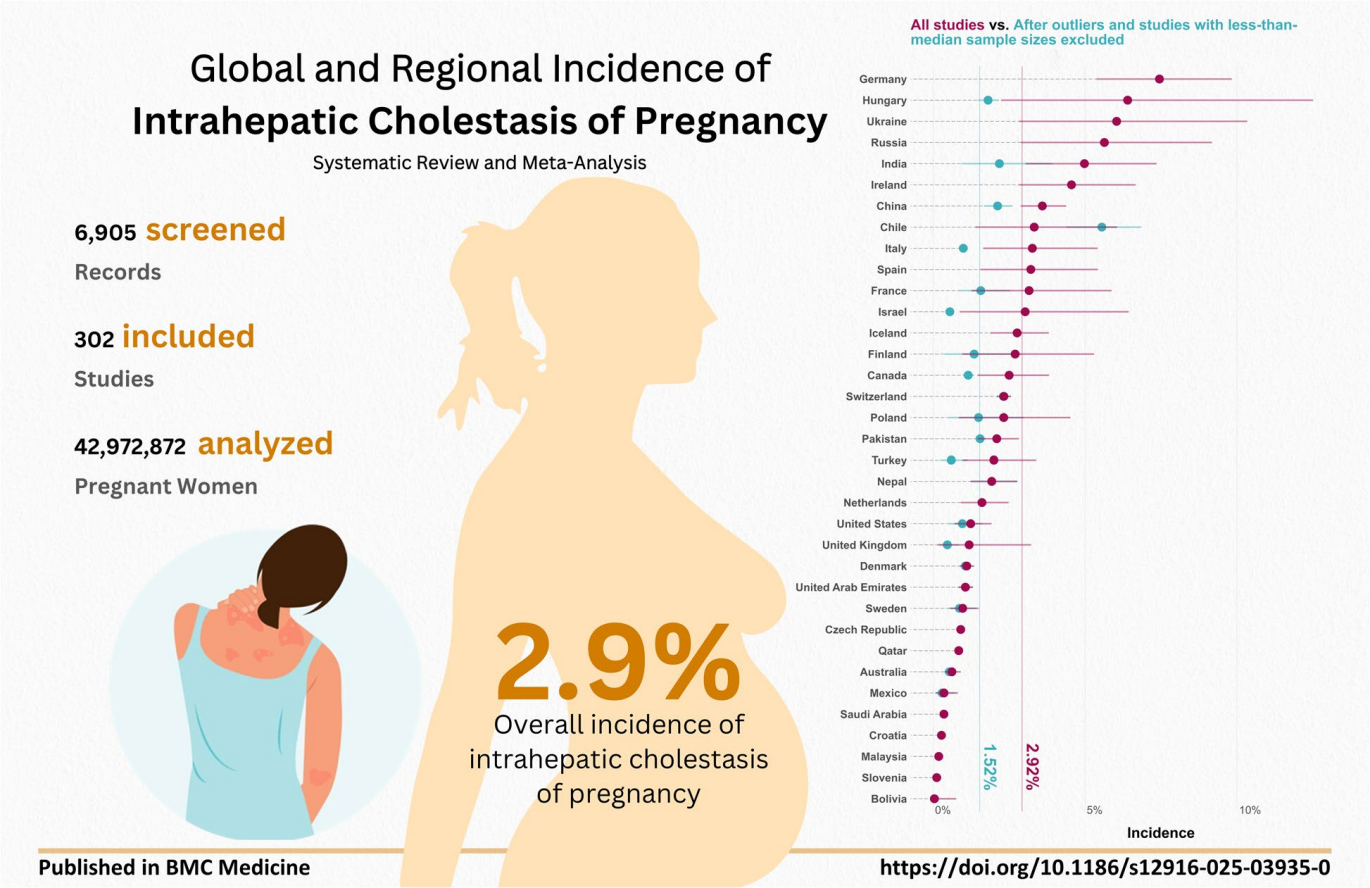

**Supplementary Information:**

The online version contains supplementary material available at 10.1186/s12916-025-03935-0.

## Background

Intrahepatic cholestasis of pregnancy (ICP) is one of the most commonly reported gestational liver diseases, which usually presents in the second and third trimesters of pregnancy. It is characterized by pruritus, which frequently subsides within a few hours or days following birth. However, biochemical alterations may persist for a longer duration due to the physiological increase in transaminases after birth, which can make diagnosis challenging or lead to misdiagnosis [1]. In patients with ICP, pruritus can markedly impair the quality of life, impose enormous distress on women with ICP, affect their well-being, and lead to suicide or psychological disorders, particularly in recurrent cases, which may necessitate several drugs, such as ursodeoxycholic acid [2]. ICP is characterized by pruritus in the absence of skin lesions [3]. Some pruritogens, such as certain lysophospholipids and sulfated progesterone metabolites, are the major mediators in the pathogenesis of cholestasis-associated pruritus [2]. However, it is important to note that pruritus may not necessarily distinguish ICP from other hepatic diseases associated with pregnancy. Other conditions such as acute fatty liver of pregnancy and severe preeclampsia can coexist with ICP, and these conditions may also present with pruritus [3]. ICP can result in maternal discomfort, increase the risk of stillbirth, spontaneous preterm birth, meconium-stained amniotic fluid, and neonatal intensive-care unit admission [4].

Although many factors, such as previous episodes of ICP, multiple pregnancies, multiple parity, low body mass index (BMI), being a hepatitis B carrier, preeclampsia, gestational hypertension (GHTN), hyperlipidemia, hepatitis C, and gestational diabetes mellitus (GDM), have been previously reported to be associated with the incidence of ICP, still there is no consensus on the risk factors of ICP based on the available evidence [1, 5–7]. On the other hand, the incidence of ICP widely varies among the current original studies from less than 1% to more than 20% [8, 9]. This considerable inconsistency among previous studies has been mostly attributed to ethnic and environmental factors. Furthermore, there is a considerable difference between reports from each country [9, 10]. For instance, according to previous reports, the incidence of ICP in the USA ranged from 0.32% in one study to 5.6% in another study [11, 12]. Similarly, the incidence of ICP was 0.62% in one study to 2% in another one from France [13, 14].

There is no meta-analysis estimating the global and regional incidence of ICP available in the literature. In this systematic review and meta-analysis, we measured the global and regional incidence of ICP and identified factors leading to variable estimates of ICP incidence in different studies.

## Methods

### Search strategy and data sources

The present review was carried out in adherence to the Preferred Reporting Items for Systematic Reviews and Meta-analyses (PRISMA) and Meta-analysis of Observational Studies in Epidemiology (MOOSE) guidelines, which are widely acknowledged as the gold standard for transparent reporting of systematic reviews and meta-analyses [15]. Furthermore, established methodological guidelines that have garnered widespread endorsement were employed to enhance the rigor and validity of the study [16, 17]. The systematic review protocol was prospectively registered in the International Prospective Register of Systematic Reviews (PROSPERO: CRD42023467481). Also, the protocol has been published elsewhere in a peer-reviewed journal [18].

The reviewers conducted a comprehensive and systematic search of PubMed, Scopus, and Web of Science using keywords and database-specific terms (MeSH) to identify pertinent studies published from the inception of these databases until July 13th, 2023. The search was updated on November 28th, 2024 using the same strategy. The detailed syntax developed for each online database is available in Additional File 1.

### Study selection and eligibility criteria

Following automatic duplicate detection, the records were uploaded to the Rayyan online tool, which is specifically designed for management of systematic reviews [19]. Three independent reviewers (PH, MAS, and NDE) screened the records by titles and abstracts. Discrepancies were resolved through discussion. The subsequent step involved a thorough assessment of the full texts to determine eligibility. Inclusion criteria presence of statistics necessary for estimating incidence of ICP and with a minimum of 50 sample participants from a representative population.

**Fig. 1:**
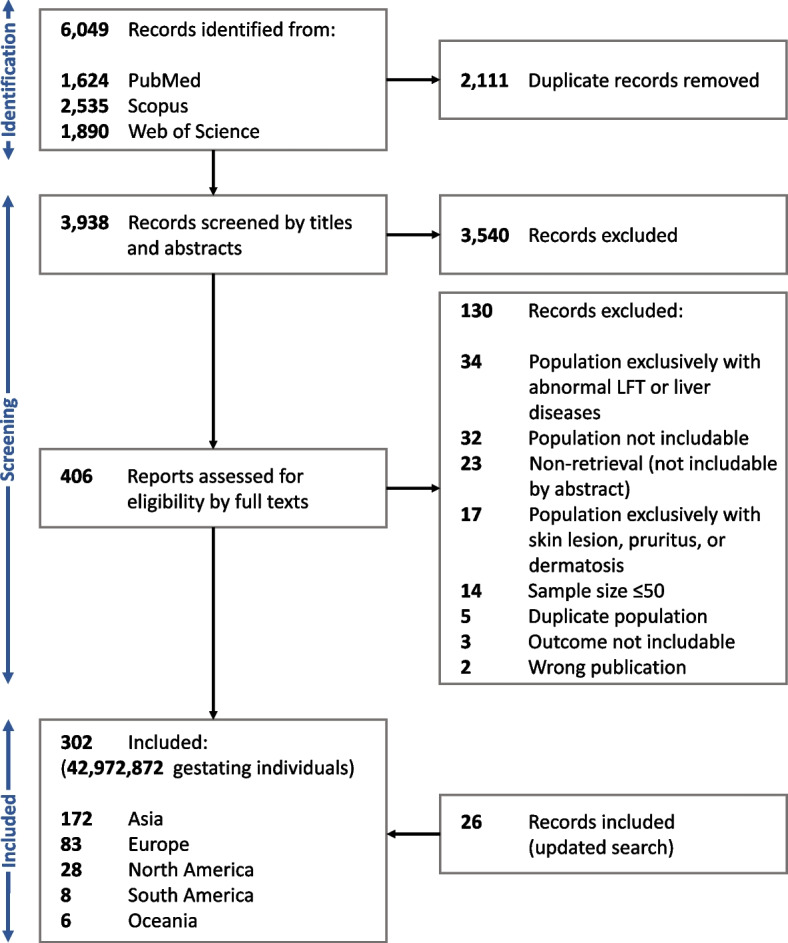
PRISMA flow chart of the literature screening. PRISMA: Preferred Reporting Items for Systematic Reviews and Meta-Analyses; LFT: Liver Function Test

To ensure an accurate estimation of the effect size, studies that tend to overestimate the incidence of ICP were excluded; these include studies that exclusively involved individuals with current or history of pruritus, hepatobiliary disorders, abnormal liver function tests, and dermatosis. To reduce language and publication bias, eligibility criteria were not restricted to English and peer-reviewed papers, therefore, grey literature were also eligible for inclusion [20]. Original articles, posters, conference abstracts, and letters to editors were considered for inclusion [21]. To prevent over-representation, only the report with the largest sample size was included in the meta-analysis when multiple studies were available on a common population of patients. A detailed PRISMA flow diagram illustrating the selection process is presented in Fig. 1.

### Risk of bias assessment and data extraction

To assess the potential for bias, Risk of Bias (RoB) was evaluated by three independent reviewers (MAS, PH, and NDE) utilizing the Joanna Briggs Institute (JBI) Critical Appraisal Checklist for incidence studies [22, 23]. For each domain, studies were scored 1 if they were considered low risk for bias. The sum of scores was used as a proxy of their overall quality.

The study-level characteristics and favorable statistics were extracted and recorded in an Excel spreadsheet. The midpoint of each study was determined by calculating the arithmetic mean of starting and finishing years of study. If no start and end times were provided, publication year was considered as midpoint. Later, studies were categorized into three distinct periods considering their midpoint: pre-2005, 2006–2015, and 2016–2023. Additionally, the countries where the studies were conducted were classified using well-established health and socioeconomic classifications from the World Health Organization (WHO), the World Bank, and the United Nations International Children’s Emergency Fund (UNICEF) based on their WHO regional classification, world bank income grouping, and continent. The country groupings can be found in Additional file 2. To estimate the distance of each country from the equator line, the absolute value of geographical latitude was utilized.

### Statistical analyses

This work was completed using R version 4.3.3 with the following R packages: camcorder v. 0.1.0, flextable v. 0.9.7, furrr v. 0.3.1.9000, future v. 1.34.0, ggforce v. 0.5.0, glue v. 1.7.0, here v. 1.0.1, janitor v. 2.2.0, magick v. 2.8.3, marquee v. 0.1.0, metafor v. 4.6.0, officer v. 0.6.7, rmarkdown v. 2.29, rnaturalearth v. 1.0.1, rnaturalearthdata v. 1.0.0, rnaturalearthhires v. 1.0.0.9000, rstatix v. 0.7.2, scales v. 1.3.0, sf v. 1.0.16, showtext v. 0.9.6, and tidyverse v. 2.0.0 [24–48]. To improve reproducibility, the scripts, data, and other materials used in this study are open source and publicly available from FigShare [49].

Statistical significance was defined as *p* < 0.05. A maximum likelihood random-effects model was utilized to combine the Freeman-Tukey double arcsine transformed effect sizes [50–52]. The Freeman-Tukey method is the preferred transformation in meta-analyses of proportions due to its reliable results [53]. After analysis, the effect sizes were back-transformed using the inverse Freeman-Tukey method. The residual between-study heterogeneity was quantified using *I*^2^ statistic [54].

To explore residual between-study heterogeneity, we applied several subgroup analyses and uni- and multivariate meta-regression modeling. Variables were first modeled in a univariate regression model. Then, ten variables with the least shared missing values were entered a brute-force feature selection approach. Models were produced using every possible combination of those variables and compared using corrected Akaike Information Criterion (AICc). Finally, the multivariate model with AICc closest to zero was selected [55, 56].

We did not evaluate publication bias in the current study, as it is not considered pertinent to incidence studies [21, 57]. However, the sensitivity of our results was examined using subgroup analysis based on the overall RoB score and peer-review status of the studies. The studies that scored five or more in RoB were compared with those that scored less than five to examine the sensitivity of the findings to potential biases. The criteria for each domain of bias used in this review are provided in Additional File3.

**Fig. 2:**
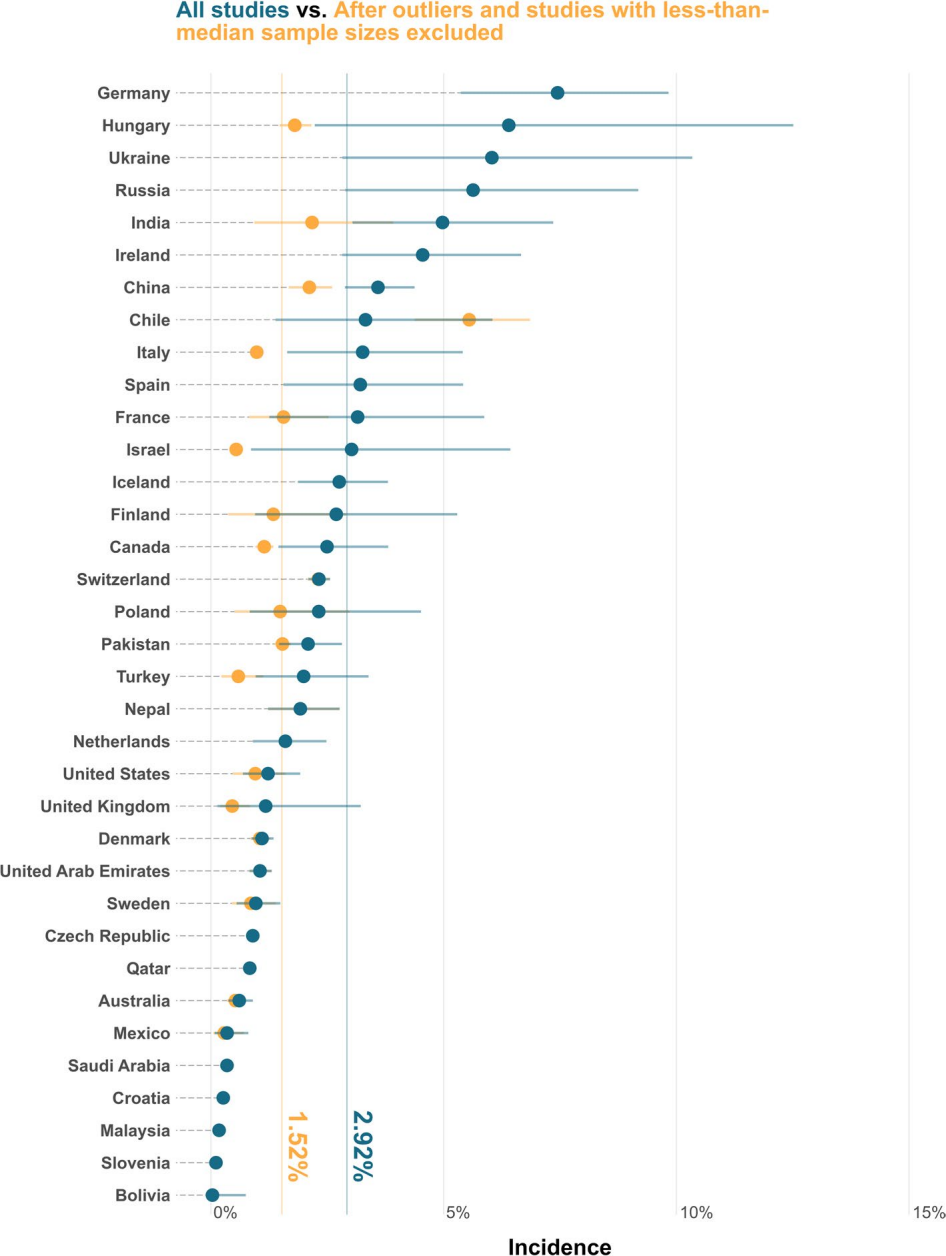
Forest plot demonstrating overall and country-level estimated incidence of ICP. Effect sizes presented in ample present results from non-outliers and those with sample sizes more than the median sample size. The effect sizes presented in cerulean present results from all the included studies. ICP: intrahepatic cholestasis of pregnancy

As findings show below, a trend of overestimation by studies with lower sample sizes is present. Therefore, every analysis, visualization, and table were produced in pairs using two distinct datasets. The first dataset was from all of the included studies, while the second focused exclusively on those with sample sizes larger than the median and effect sizes that were not considered outliers. The results of the analyses based on the second dataset are available from Fig. 2 and Additional Files 4–6.

## Results

The search generated 6049 records on the first round, of which, 2111 were found to be duplicates using automatic tools. Of the remaining, 276 studies with a total number of 32,401,818 pregnant women were included in the evidence synthesis. The updated search led to inclusion of 26 additional records with the sum of 10,571,054 individual pregnant women. The included studies were published from 35 countries. One hundred ninety seven studies were from Asia, 83 from Europe, 28 from North America, 8 from South America, and 6 from Oceania (Fig. 1). The setting for all of the studies was medical centers. The data for included studies are provided in Additional File 2 [5–8, 10–12, 14, 58–326]. The following citations were entered the study after search was updated: [327–352].

The global pooled incidence of ICP and its 95% confidence interval (CI) was 2.9% [2.5, 3.3] from all the studies and 1.5% from non-outlier studies with larger-than-median sample sizes. The subgroup analyses showed that South-East Asian Region (SEAR) had the highest incidence of ICP 4.7% [2.9%, 6.9%] while the incidence was the lowest in the Eastern Mediterranean Region (EMR) 1.6% [1.1%, 2.3%] among the WHO regional classes. Other regions had the following incidence: Western Pacific Region (WPR) 3.4% [2.7%, 4.1%], European Region (EUR) 2.5% [2.0%, 3.2%], and Americas (AMR) 1.6% [1.0%, 2.2%]. Figure 2 and Table 1 demonstrate ICP incidence for different classifications, regions, and countries (see Additional File 5 for the same analysis using non-outlier studies with larger-than-median sample sizes only).

**Table 1:**
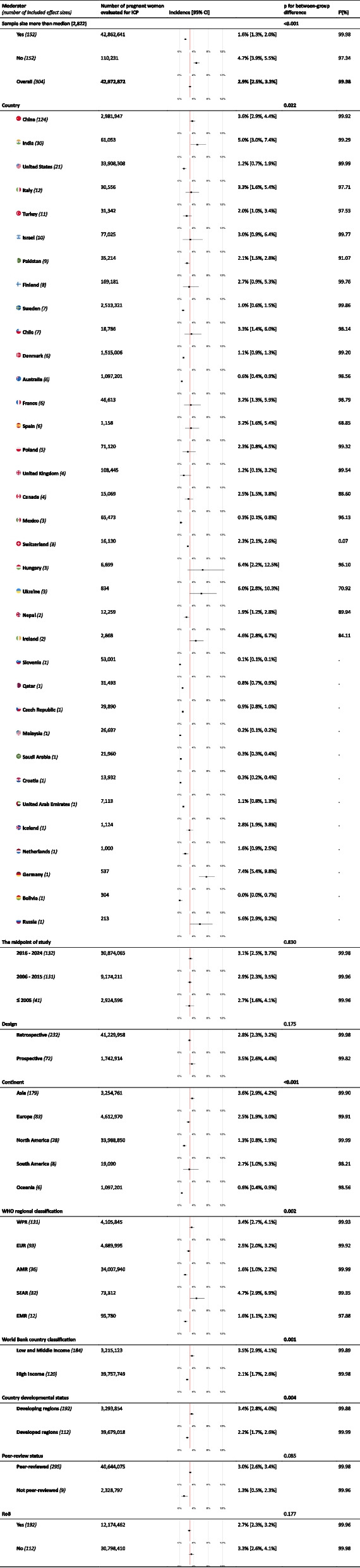
Moderator analysis of the pooled incidence of ICP

Single effect size forest plots show the pooled effect size in that subgroup and the purple line shows the overall pooled effect size

The cut-off was set at the median of sample sizes across the included studies

Note that this table presents the overall pooled estimates from all of the included studies. However, studies with lower sample sizes tend to overestimate the incidence and introduce bias. Therefore, readers who are interested in the estimates from studies with greater sample sizes only (more than the median sample size of all the included studies in this table: 2822) are recommended to consult with Additional File 5.

*p* values faced with bold are less than the statistical significance threshold (0.05)

*ICP* Intrahepatic Cholestasis of Pregnancy, *CI* Confidence Interval, *WHO* World Health Organization, *AMR* Region of the Americas, *EMR* Eastern Mediterranean Region, *EUR* European Region, *WPR* Western Pacific Region, *SEAR* South-East Asian Region, *RoB* Risk of Bias

As shown in Table 1, our subgroup analysis of studies midpoint (cut-offs: 2005 and 2015) was not statistically significant (*p* = 0.83). This finding was further supported with univariate random-effect meta-regression (Table 2), indicating the absence of a linear or polytomous temporal pattern of ICP incidence (*p* = 0.12). However, when we removed outlier studies and those with sample sizes smaller than the median sample size, an increasing pattern surfaced in both subgroup analyses: incidence rose from 1.0% [0.5%, 1.6%] in midpoints before 2005 to 1.4% [1.0%, 1.8%] in 2006–2015, and up to 1.8% [1.5%, 2.3%] in 2016–2024 (*p* = 0.04) (Additional File 5).

**Table 2:** Multiple univariate and multivariate meta-regression analysis

**Term**	**Estimate [95% CI]**	***p* value**	**Estimate [95% CI]**	***p* value**
Multiple pregnancies	0.08 [0.04, 0.12]	** < 0.001**	0.09 [0.02, 0.16]	**0.014**
GDM	0.23 [0.09, 0.37]	**0.001**	0.43 [0.04, 0.82]	**0.030**
Nulliparous	0.08 [−0.01, 0.16]	0.084	0.11 [−0.01, 0.23]	0.072
Mean maternal age	−0.00 [−0.01, 0.00]	0.215	−0.01 [−0.02, 0.00]	0.113
Absolute latitude	−0.00 [−0.00, 0.00]	0.211	−0.00 [−0.01, 0.00]	0.272
Study end year	0.00 [−0.00, 0.00]	0.131	−0.00 [−0.01, 0.00]	0.317
RoB score	−0.01 [−0.02, 0.00]	0.154	−0.00 [−0.04, 0.04]	0.890
GHTN	0.69 [0.38, 1.01]	** < 0.001**		
Preterm labor	0.15 [0.07, 0.23]	** < 0.001**		
PROM	0.38 [0.14, 0.63]	**0.002**		
Vaginal delivery	−0.13 [−0.23, −0.03]	**0.009**		
Hepatitis C	0.09 [0.00, 0.17]	**0.038**		
Study start year	0.00 [−0.00, 0.00]	0.069		
Study midpoint	0.00 [−0.00, 0.00]	0.123		
Preeclampsia	0.08 [−0.03, 0.18]	0.142		
Smoker	−0.49 [−1.18, 0.20]	0.162		
Stillbirth	−0.05 [−0.12, 0.03]	0.231		
DM	0.80 [−0.52, 2.12]	0.234		
Mean parity	−0.07 [−0.21, 0.07]	0.323		
Publication year	0.00 [−0.00, 0.00]	0.355		
HTN	−0.17 [−0.83, 0.50]	0.626		
HBS	0.05 [−0.16, 0.25]	0.667		
ART	0.00 [−0.05, 0.06]	0.869		
Mean BMI	0.00 [−0.01, 0.01]	0.902		
Undereducation	−0.00 [−0.31, 0.30]	0.980		

Multiple independent univariate regression models were produced. Brute-force approach was used to model all possible combinations of ten moderators with the least shared missing values. Then, the best model was chosen using AICc

*p* values faced with bold are less than the statistical significance threshold (0.05)

A similar table presenting results of meta-regression when outlier studies and those that have sample sizes less than median were excluded is available in Additional File 10

*AICc* Corrected Akaike Information Criterion, *CI* Confidence Interval, *GHTN* Gestational Hypertension, *GDM* Gestational Diabetes Mellitus, *PROM* Premature Rupture of Membrane, *DM* Diabetes Mellitus, *HTN* Hypertension, *BMI* Body Mass Index, *ART* Assisted Reproductive Technology

**Fig. 3:**
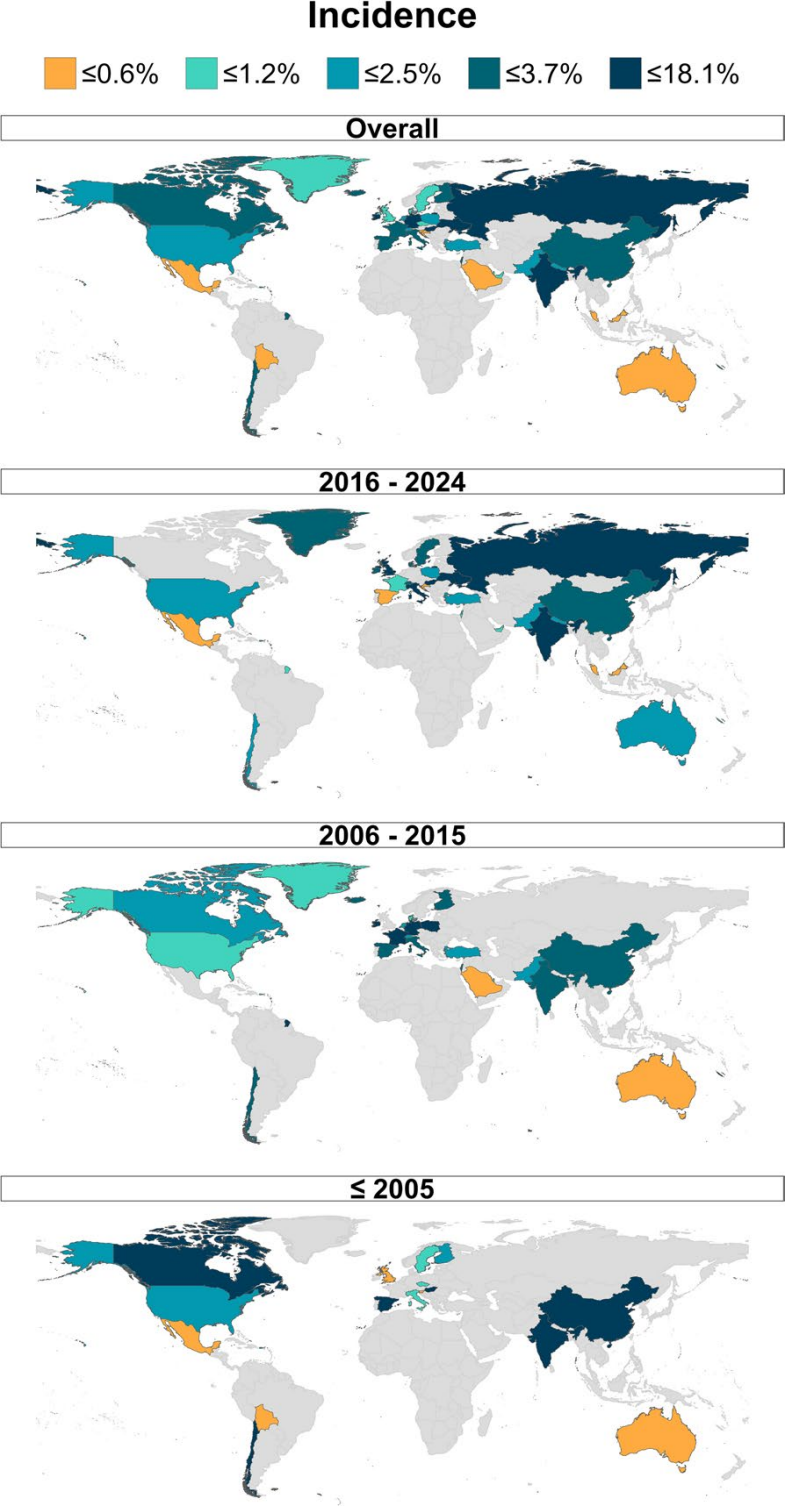
Choropleth demonstrating the time trend of incidence of ICP. ICP: intrahepatic cholestasis of pregnancy

Further subgroup analyses were done to investigate the potential effects of moderators on the incidence of ICP. Prospective and retrospective studies did not show different overall estimates (*p* = 0.17). Studies with sample sizes larger than 2822 (median sample size of the included studies) showed a lower incidence of ICP 1.6% [1.3, 2.0], compared to those with smaller sample sizes 4.7% [3.9, 5.5] (*p* < 0.001), indicating a trend of overestimation by studies with lower sample sizes. Therefore, two iterations of the meta-analysis were conducted: the first included all studies (Figs. 2 and 3 and Tables 1 and 2), while the second included only those studies with sample sizes larger than 2822 (Fig. 2 and Additional Files 4–6).

We also evaluated the effects of World Bank grouping and the developmental status of countries as potential socioeconomic moderators of the incidence of ICP. Countries with low and middle income 3.5% [2.9%, 4.1%] and developing economies had a higher incidence of ICP compared to high-income countries 2.1% [1.7%, 2.6%] (*p* = 0.001).

Uni- and multivariate meta-regression modeling (Table 2) both showed that the incidence of ICP was positively associated with proportion of pregnant women with (*β* coefficient and its 95% CI for multivariate estimate): multiple pregnancies 0.09 [0.02, 0.16] (*p* = 0.01) and GDM 0.43 [0.04, 0.82] (*p* = 0.03). Also, GHTN 0.69 [0.38, 1.01] (*p* < 0.001), preterm labor 0.15 [0.07, 0.23] (*p* < 0.001), Premature Rupture of Membrane (PROM) 0.38 [0.14, 0.63] (*p* = 0.002), vaginal delivery − 0.13 [− 0.23, − 0.03] (*p* = 0.009), and hepatitis C 0.09 [0.00, 0.17] (*p* = 0.03) showed significant associations with ICP incidence in univariate regression analysis. Meta-regression results using the non-outlier studies and studies with larger-than-median sample sizes are provided in Additional File 6.

The sensitivity of our results was examined using subgroup analyses based on the RoB score and peer-review status of the studies (Table 1). Studies considered low RoB (scored considered low RoBts was examined using subgrogh RoB (*p* = 0.17). Also, *p* value for between-group difference was not significant for peer-reviewed versus not peer-reviewed studies (*p* = 0.085). However, it should be noted that the not-peer-reviewed group only consisted of 9 records, whereas 295 records were peer-reviewed. The overall evaluation for each domain of bias is available from Additional File7. Also, the reviewers’ detailed assessment of RoB for each study is available from Additional File2.

The JBI tool employed in this study includes a criterion that views sample size as a potential bias source. It employs a method suggested by Naing and colleagues [353]. This method takes a single study only into account to establish an evaluation cut-off. However, the researchers argued that when setting a minimum sample size as an evaluation cut-off for RoB, all studies should be taken into account. As a result, the median of all study sample sizes was selected as the cut-off for sample sizes as their values are highly skewed toward larger sample sizes. However, to maintain fidelity to the primary tool, its criteria are also reported unaltered in Additional File2. It is noteworthy that pooled estimations from studies with higher RoB scores did not statistically differ from those from studies with lower scores, which is likely because a single scale, reduced from nine distinct bias domains (sum of all scores), is insufficient for classifying the studies [354].

## Discussion

This systematic review and meta-analysis indicated that ICP is detectable in 2.9% [2.5%, 3.3%] of pregnant women worldwide; however, the incidence widely differs across countries, with Germany, Hungary, Ukraine, Russia, India, Ireland, and China having the highest incidence and Bolivia, Slovenia, Malaysia, Croatia, Saudi Arabia, Mexico, and Australia having the lowest incidence of ICP. In addition, a lower incidence of ICP was found in high-income and developed countries. Among the continents, the incidence of ICP was higher in South America and Asia while lower in Oceania and North America. Among WHO regions, the pooled incidence was markedly lower in AMR and EMR and higher in SEAR. There was no significant difference between prospective and retrospective studies. Interestingly, after excluding outliers and studies whose sample size was less than the median sample size, the incidence of ICP decreased from 2.9 to 1.5%, indicating an overestimation by lower sample sizes. By removing outliers and smaller sample sizes, a trend of gradual increase appears over time, with a pooled incidence of 1.0% before 2005 and an incidence of 1.8% in 2016–2024. Multivariate meta-regression model suggests a positive association between the proportion of women with multiple pregnancies and GDM with the incidence of ICP.

ICP is one of the most common gestational liver diseases that may lead to adverse outcomes [3, 285, 355]. For instance, previous studies have found that ICP is linked to GDM, hyperlipidemia, preterm delivery, pre-eclampsia, stillbirth, neonatal unit admission, and meconium-stained amniotic fluid [4, 7, 285, 356]. Furthermore, ICP can markedly impair the quality of life of pregnant women and impose a great psychological burden [2]. On the other hand, the reported incidence of ICP comprises a wide range and there is no consistency between previous studies concerning the incidence, predictors, and consequences of ICP [7, 285, 356, 357]. Therefore, a meta-analysis providing sufficient information on the incidence and potential predictors and consequences of ICP is indispensable to appropriately allocate resources and design guidelines for managing this medical condition.

This meta-analysis indicated that the incidence of ICP widely varies between different countries, continents, and WHO regions. Besides, individual studies have shown that the incidence of ICP differs considerably between various ethnic groups in each country [10–12]. These findings suggest the need for region-specific guidelines to more effectively screen and manage ICP in each region.

Some studies also reported that a past ICP history can predict a markedly increased risk of future ICP [59]. Interestingly, a nationwide Danish study reported that the incidence of ICP is much higher among co-twins and first-degree relatives of women who experience ICP [358]. The study also indicated that the first-degree relatives of women who experienced recurrent ICP or first-trimester ICP had a higher risk of the disease [358]. Together, these findings support the critical role of ethnicity and genetic background in the development of ICP [10–12].

Homozygous variants of *ABCB4* and *ABCB11* genes, bile constituent transporters, can lead to severe cholestasis. In addition, their heterozygous variants increase the risk of ICP and are believed to be involved in > 20% of ICP cases [359]. To further highlight the genetic background, genome-wide association studies have shown the potential role of different variants of bile acid homeostasis genes, such as *CYP7A1* and *SULT2A1*, cellular transporter genes, and other genes in the pathogenesis of ICP [360]. Particularly, variations in liver-specific genes and *cis*-regulatory elements were shown to potentially increase susceptibility to ICP [360]. Interestingly, the study developed a polygenic risk score model for ICP, which indicated that patients with ICP have a significantly higher burden of cholelithiasis genetic risk variants compared to control subjects [360]. Moreover, it has been hypothesized that increased levels of reproductive hormones can unmask genetic susceptibility to ICP in some women [361].

Consistent with the role of estrogen in inducing ICP, meta-regression uncovered that multiple pregnancies are associated with a higher incidence of ICP. In addition, meta-regression indicated that ICP is positively associated with GDM and preterm labor and negatively associated with vaginal delivery. Consistently, due to the high risk of complications, such as stillbirth, the latest guidelines recommend preterm induction of labor in those with ICP and total serum bile acid concentrations ≥ onceμmol/l [362]. In particular, it has been previously observed that compared with late-onset ICP, early-onset ICP is associated with a higher risk of preterm labor, fetal distress, and fetal low birth weight [180]. Thus, either as a complication or as part of the treatment procedure, ICP can increase the risk of preterm delivery.

Subgroup analyses of both datasets indicate that the incidence of ICP is negatively correlated with income and development status. In other words, developed countries with higher income levels tend to report lower incidences of ICP. This trend may be reflective of more advanced public and maternal health systems in these nations, which contribute to a reduced ICP incidence. Conversely, some may contend that these findings are influenced by potential overestimations and variability in reporting quality from smaller studies from developing and low-income countries. Arguably, the presence of comprehensive, high-quality registries in developed countries skews the data, suggesting that these differences may not accurately reflect real-world disparities in ICP incidence between developed and developing nations. However, a counterargument arises when examining data from non-outlier studies with larger-than-median sample sizes, which consistently show similar trends (Additional File 5). Therefore, the authors contend that it is reasonable to conclude that ICP incidence is indeed lower in developed and high-income countries, reflecting the broader effects of improved maternal health and healthcare access on the incidence of ICP.

Given the significant differences between regions and ethnicities in the incidence of ICP and the critical consequences of this medical condition, future large-scale prospective studies should identify the factors predicting ICP and unravel the genetic basis of this medical condition. We identified the associations between ICP and some clinical features, which can help clinicians promote awareness of ICP in specific groups of patients. However, we could not establish a causal relationship due to the nature of the included studies.

### Strengths and limitations

To the best of our knowledge, this is the first study that estimates a global and regional incidence of ICP. With a deliberate effort to mitigate language and publication biases, the study chose not to impose specific constraints on eligibility criteria, such as language, publication time, or publication type. Additionally, sensitivity analyses were conducted to assess the potential impact of these factors on the final estimation.

However, it is crucial to acknowledge certain limitations inherent in our research. Although our investigation succeeded in identifying studies from 35 countries worldwide, a significant number of countries remain unaccounted for in estimating the incidence of ICP. Consequently, there exists an inherent incomplete geographical representation, particularly in developing and underdeveloped nations. Furthermore, the studies included in our analysis exhibited substantial variation in terms of time period, methodology, sample population, definitions, and identification methods, thus contributing to notable heterogeneity. This heterogeneity, observed even among populations sharing similar characteristics, hints at local disparities in the risk factors associated with ICP. Regrettably, as seen in many systematic reviews of incidence, our study did not unveil the precise sources of this heterogeneity [17, 363–365]. The current study investigates geographical coordinates of each country’s capital as potential sources of heterogeneity. However, it is important for readers to understand that many countries cover extensive areas and some, such as Chile, have irregular shapes. Therefore, findings related to these aspects should be interpreted with these considerations in mind. Also, we were not able to investigate the effects of seasonal variations, advanced maternal age, and selenium and vitamin D deficiency on the incidence of ICP due to the scarcity of available data. It should be noted that the current study was not designed to detect epidemiologic trends over time. As most of the included studies were conducted on extended time spans, pooling such study-level time trends introduces challenge to analysis.

## Conclusions

The pooled incidence of ICP was 2.9% among all studies, which decreased to 1.5% after excluding outliers and studies whose sample sizes were less than the median sample size (Fig. 2). The incidence was higher in South America and Asia and developing and low- and middle-income countries. On the contrary, the incidence was lower in Oceania, North America, and Europe. At the country level, the incidence widely differed across countries, with Germany, Hungary, Ukraine, Russia, India, Ireland, and China having the highest incidence and Bolivia, Slovenia, Malaysia, Croatia, Saudi Arabia, Mexico, and Australia having the lowest incidence of ICP, respectively. We also found that multiple pregnancies, preterm labor, hepatitis C, GHTN, GDM, and PROM were positively associated with the incidence of ICP. In contrast, vaginal delivery was inversely associated with the incidence of ICP. These findings may help health policy-makers to adjust resource allocation and management guidelines in the future. As evident from the results, small sample sizes tend to overestimate the incidence. Therefore, for future research focused on a comprehensive evaluation of ICP epidemiology, it is advisable to adjust the sample estimates accordingly. Readers are strongly encouraged to consult with additional files, which include findings from non-outlier studies with sample sizes greater than the median.

## Supplementary Information

Additional File 1. Search Strategy of the first and second searches.

Additional File 2. Study level data used to produce the results.

Additional File 3. Criteria employed to assess the risk of bias.

Additional File 4. Choropleth demonstrating the time trend of incidence of Intrahepatic Cholestasis of Pregnancy. This plot only uses non-outlier studies with larger-than-median sample sizes.

Additional File 5. Table of subgroup analysis. This table only uses non-outlier studies with larger-than-median sample sizes.

Additional File 6. Table of meta-regression. This table only uses non-outlier studies with larger-than-median sample sizes.

Additional File 7. Stacked bar plot of judgments about each risk of bias item presented as proportions across all included studies.

## Data Availability

The data underlying this article are available in the article and its online supplementary material. Also, data and code are stored in an online repository: Sadeghi, Alireza; Dehdari Ebrahimi, Niloofar; Jamshidi Kerachi, Ali; Shahlaee, Mohammad Amin; Habibi, Pardis (2024). Data and material used to produce results for the meta-analysis of the incidence of cholestasis of pregnancy. figshare. Dataset. https://doi.org/10.6084/m9.figshare.28010117.v2.

## Abbreviations

AMR: Region of the Americas
ART: Assisted reproductive technology
BMI: Body mass index
CI: Confidence interval
EMR: Eastern Mediterranean Region
EUR: European Region
GDM: Gestational diabetes mellitus
GHTN: Gestational hypertension
ICP: Intrahepatic cholestasis of pregnancy
JBI: Joanna Briggs Institute
PRISMA: Preferred Reporting Items for Systematic Reviews and Meta-analyses
PROM: Premature rupture of membrane
RoB: Risk of bias
SEAR: South-East Asian Region
UNICEF: United Nations International Children’s Emergency Fund
WHO: World Health Organization
WPR: Western Pacific Region
