# Supplementary material for: Global and regional incidence of intrahepatic cholestasis of pregnancy: a systematic review and meta-analysis
Source: BMC Med. 2025 Feb 28;23:129. doi: 10.1186/s12916-025-03935-0 (PMC11871686; doi:10.1186/s12916-025-03935-0)
Supplement: Supplementary file 1 — Additional File 1. Search Strategy of the first and second searches. [file 12916_2025_3935_MOESM1_ESM.pdf]

1 Search strategy

Date first search was done: July 13th, 2023

Date first search was done: November 28th, 2024

1.1 PubMed

("pregnancy"[Title/Abstract] OR "pregnant"[Title/Abstract] OR "gestational"[Title/Abstract] OR "pregnancy"[MeSH Terms]) AND ("cholestasis"[Title/Abstract] OR "cholestatic"[Title/Abstract] OR (("biliary"[Title/Abstract] OR "hepatobiliary"[Title/Abstract] OR "bile duct"[Title/Abstract]) AND ("obstruction"[Title/Abstract] OR "stasis"[Title/Abstract])) OR "cholestasis, intrahepatic"[MeSH Terms]) AND ("intrahepatic"[Title/Abstract] OR "intra-hepatic"[Title/Abstract])

first search: 1,624, second search: 208

1.2 Scopus

TITLE-ABS-KEY ("pregnancy" OR "pregnant" OR "gestational") AND TITLE-ABS-KEY ("cholestasis" OR "cholestatic" OR (("biliary" OR "hepatobiliary" OR "bile duct") AND ("obstruction" OR "stasis"))) AND TITLE-ABS-KEY ("intrahepatic" OR "intra-hepatic")

first search: 2,535, second search: 434

1.3 Web of Science

TS=("pregnancy" OR "pregnant" OR "gestational") AND TS=("cholestasis" OR "cholestatic" OR (("biliary" OR "hepatobiliary" OR "bile duct") AND ("obstruction" OR "stasis"))) AND TS=("intrahepatic" OR "intra-hepatic")

first search: 1,890, second search: 214
