# Supplementary material for: Global and regional incidence of intrahepatic cholestasis of pregnancy: a systematic review and meta-analysis
Source: BMC Med. 2025 Feb 28;23:129. doi: 10.1186/s12916-025-03935-0 (PMC11871686; doi:10.1186/s12916-025-03935-0)
Supplement: Supplementary file 3 — Additional File 3. Criteria employed to assess the risk of bias. [file 12916_2025_3935_MOESM3_ESM.pdf]

1 Criteria employed in assessment of risk of bias

We employed Joanna Briggs Institute (JBI) Critical Appraisal Tool for appraisal of prevalence studies. The criteria that were used by the reviewers to assess the risk of bias in each domain of bias are provided here.

Domain 1. Sample frame

The studies were considered low risk for bias in sample frame if they have recruited healthy pregnant women and healthy newborns for evaluation of intrahepatic cholestasis of pregnancy.

Domain 2. Sampling method

The studies were considered low risk for bias in sample frame if they have recruited their sample participants utilizing a census, consecutive sampling, random sampling (should have reported the method clearly). The studies that recruited non-random sampling were considered high risk of bias in this domain. Also, the studies that did not describe the sampling method were considered unclear for risk of bias.

Domain 3. Sample size

Using the method proposed by Naing et al (301) and data provided by Gao et al (390), we decided that an adequate sample size should be at least 88 for a study to be considered low risk of bias in this domain.

Domain 4. Description of setting

We considered studies that have reported age of the participants (mean and standard deviation OR median and range and/or interquartile range), timeframe of the study, geographical location, and health status of the participants as low risk of bias in this domain.

Domain 5. Coverage of statistical analysis

Studies were considered low risk for bias if they have covered all of the included participants in the analyses.

Domain 6. Identification method

We used the recent *Royal College of Obstetricians and Gynaecologists'*s guideline as the appropriate method of identification of intrahepatic cholestasis of pregnancy. Accordingly, studies that used serum bile acid (>19 mmol/l) and itching (with normal skin and no rashes) to identify intrahepatic cholestasis of pregnancy were considered low risk for bias in this domain.

Domain 7. Standard and reliable measurement

If all of the sample participants were covered in the measurements and the standard methods were used to identify the condition, the studies were considered low risk for bias in this domain.

Domain 8. Statistical analysis

The numerator and denominator should be clearly reported in a study to be considered low risk of bias in this domain.

Domain 9. Response rate

The authors should have clearly discussed the response rate and any reasons for non-response and compared persons in the study to those not in the study, particularly with regards to their socio-demographic characteristics.
