# Supplementary figures and images for: Global and regional incidence of intrahepatic cholestasis of pregnancy: a systematic review and meta-analysis
Source: BMC Med. 2025 Feb 28;23:129. doi: 10.1186/s12916-025-03935-0 (PMC11871686; doi:10.1186/s12916-025-03935-0)

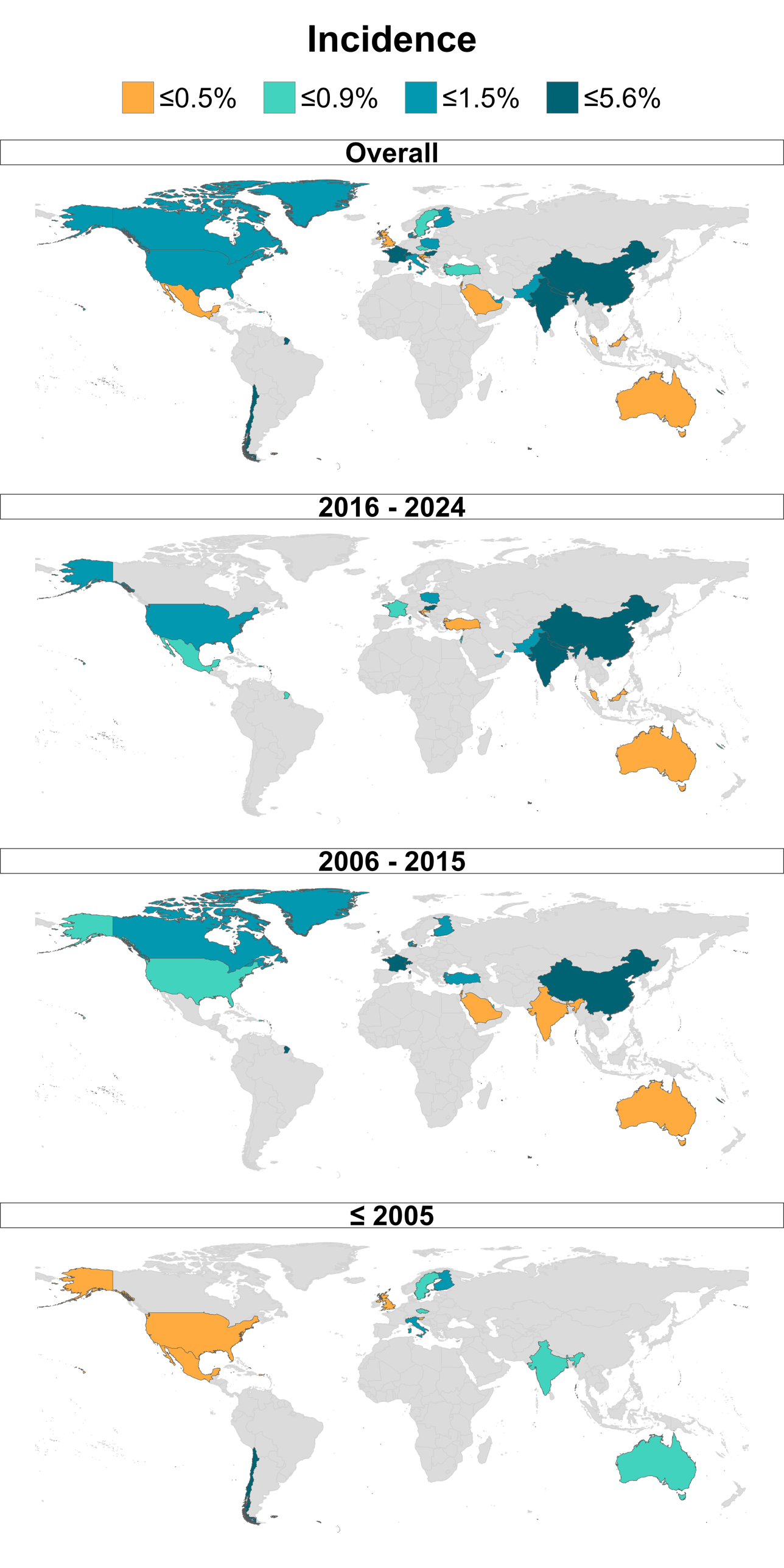

Supplement: Supplementary file 4 — Additional File 4. Choropleth demonstrating the time trend of incidence of Intrahepatic Cholestasis of Pregnancy. This plot only uses non-outlier studies with larger-than-median sample sizes. [file 12916_2025_3935_MOESM4_ESM.png]

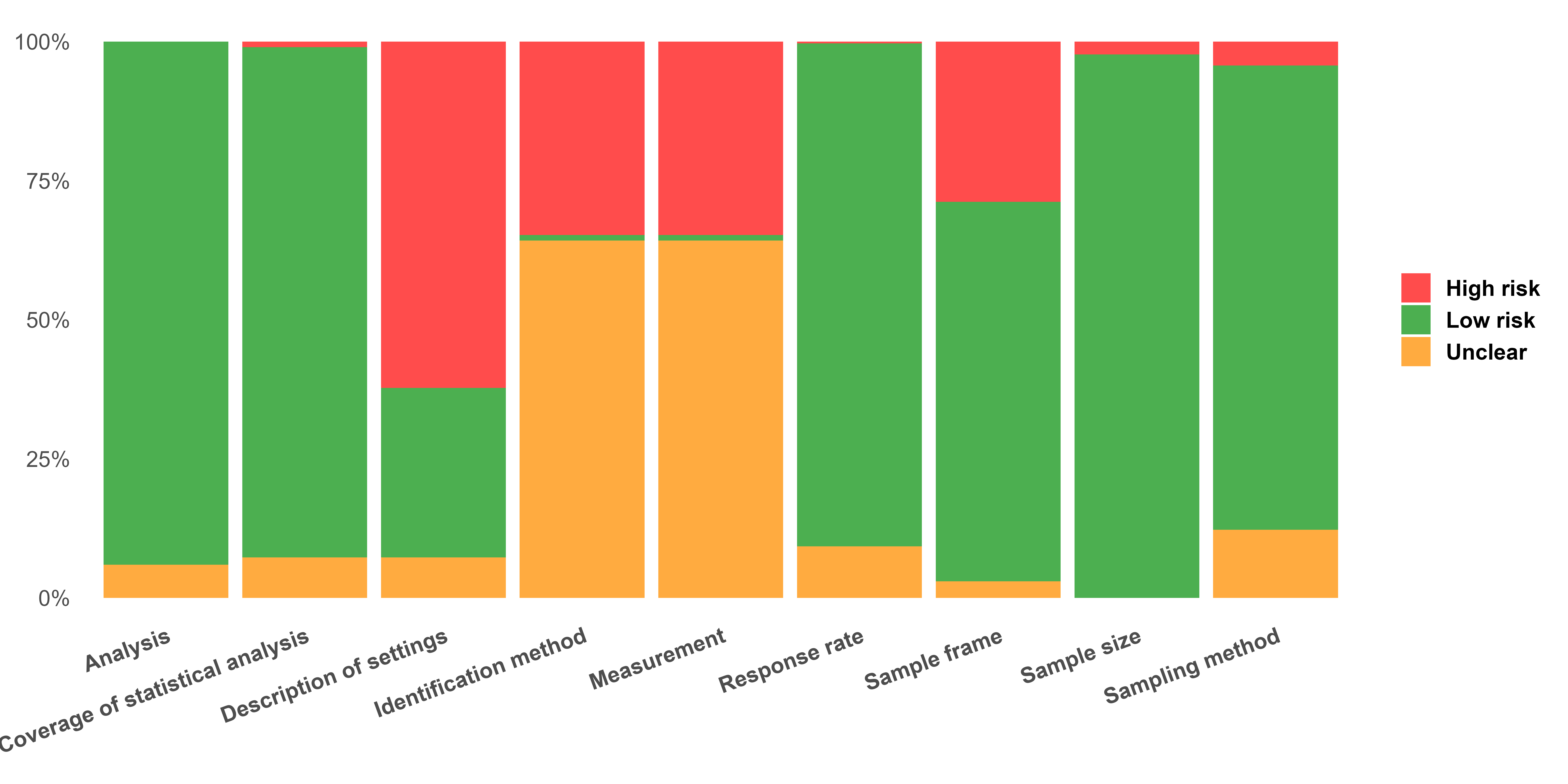

Supplement: Supplementary file 7 — Additional File 7. Stacked bar plot of judgments about each risk of bias item presented as proportions across all included studies. [file 12916_2025_3935_MOESM7_ESM.png]
