# Supplementary material for: Global and regional incidence of intrahepatic cholestasis of pregnancy: a systematic review and meta-analysis
Source: BMC Med. 2025 Feb 28;23:129. doi: 10.1186/s12916-025-03935-0 (PMC11871686; doi:10.1186/s12916-025-03935-0)
Supplement: Supplementary file 5 — Additional File 5. Table of subgroup analysis. This table only uses non-outlier studies with larger-than-median sample sizes. [file 12916_2025_3935_MOESM5_ESM.pdf]

Table Sup. 1. Moderator analysis of the pooled incidence of ICP using non-outlier studies with sample sizes larger than the median sample size.

Moderator *(number of included effect sizes)*	Number of pregnant women evaluated for ICP	Incidence [95% CI]	p for between-group difference	I²(%)
**Sample size more than median (2,822)**				
**Yes** *(150)*	**42,842,683**	**1.5% [1.3%, 1.8%]**		**99.97**
**Overall** *(150)*	**42,842,683**	**1.5% [1.3%, 1.8%]**		**99.97**
**Country**			**0.002**	
**China** *(69)*	2,906,850	2.1% [1.7%, 2.6%]		99.87
**United States** *(16)*	33,903,949	1.0% [0.5%, 1.6%]		99.99
**India** *(9)*	52,326	2.2% [0.9%, 3.9%]		99.28
**Denmark** *(5)*	1,514,718	1.1% [0.8%, 1.3%]		99.24
**Australia** *(5)*	1,096,923	0.5% [0.3%, 0.7%]		98.16
**Finland** *(5)*	167,523	1.3% [0.4%, 2.9%]		99.67
**Sweden** *(4)*	2,511,965	0.9% [0.5%, 1.4%]		99.92
**Israel** *(4)*	72,010	0.5% [0.4%, 0.7%]		78.86
**United Kingdom** *(3)*	107,673	0.5% [0.2%, 0.8%]		96.20
**Poland** *(3)*	69,195	1.5% [0.5%, 3.0%]		99.12
**France** *(3)*	46,269	1.6% [0.8%, 2.5%]		96.74
**Pakistan** *(3)*	31,137	1.5% [1.4%, 1.7%]		26.54
**Turkey** *(3)*	25,103	0.6% [0.2%, 1.1%]		93.43
**Mexico** *(2)*	65,377	0.3% [0.1%, 0.7%]		97.82

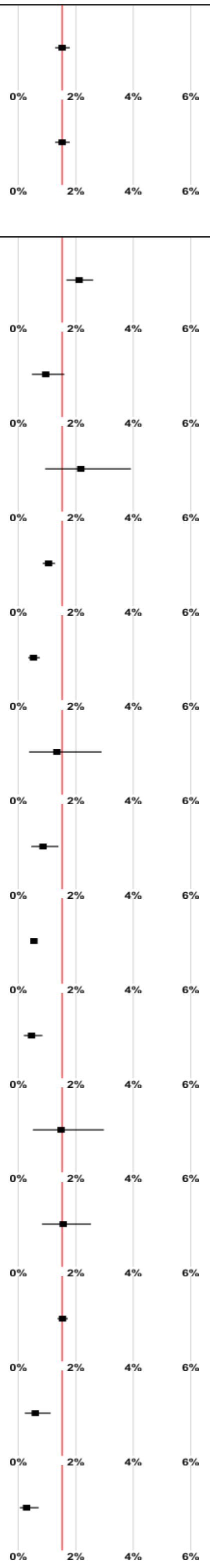

Table Sup. 1. Moderator analysis of the pooled incidence of ICP using non-outlier studies with sample sizes larger than the median sample size.

**Moderator** *(number of included effect sizes)*	**Number of pregnant women evaluated for ICP**	**Incidence [95% CI]**	**p for between-group difference**	**I²(%)**
**Italy** *(2)*	26,655	1.0% [0.9%, 1.1%]		0.00
**Chile** *(2)*	15,623	5.6% [4.4%, 6.9%]		90.30
**Nepal** *(2)*	12,259	1.9% [1.2%, 2.8%]		89.94
**Slovenia** *(1)*	53,001	0.1% [0.1%, 0.1%]		.
**Qatar** *(1)*	31,493	0.8% [0.7%, 0.9%]		.
**Czech Republic** *(1)*	29,890	0.9% [0.8%, 1.0%]		.
**Malaysia** *(1)*	26,697	0.2% [0.1%, 0.2%]		.
**Saudi Arabia** *(1)*	21,960	0.3% [0.3%, 0.4%]		.
**Switzerland** *(1)*	15,083	2.3% [2.1%, 2.6%]		.
**Croatia** *(1)*	13,932	0.3% [0.2%, 0.4%]		.
**Canada** *(1)*	12,132	1.1% [1.0%, 1.3%]		.
**United Arab Emirates** *(1)*	7,113	1.1% [0.8%, 1.3%]		.
**Hungary** *(1)*	5,827	1.8% [1.5%, 2.2%]		.
**The midpoint of study**			**0.042**	
**2016 - 2024** *(73)*	30,816,425	1.8% [1.5%, 2.3%]		99.97
**2006 - 2015** *(56)*	9,111,768	1.4% [1.0%, 1.8%]		99.96
**≤ 2005** *(21)*	2,914,490	1.0% [0.5%, 1.6%]		99.93
**Design**			0.385	
**Retrospective** *(121)*	41,124,502	1.5% [1.2%, 1.8%]		99.98
**Prospective** *(29)*	1,718,181	1.8% [1.2%, 2.5%]		99.82

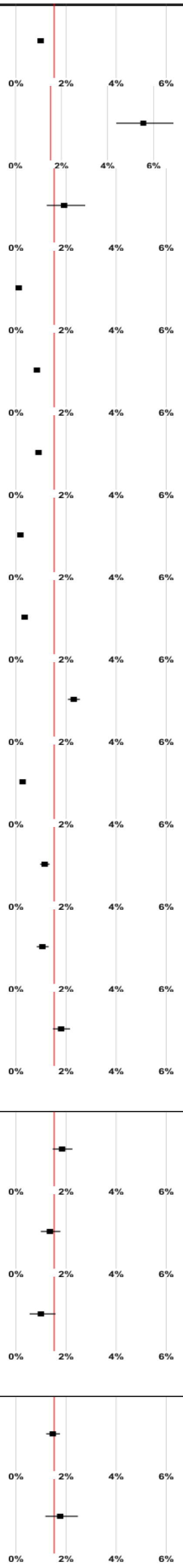

Table Sup. 1. Moderator analysis of the pooled incidence of ICP using non-outlier studies with sample sizes larger than the median sample size.

Moderator *(number of included effect sizes)*	Number of pregnant women evaluated for ICP	Incidence [95% CI]		p for between-group difference	I^2(%)
**Continent**				**<0.001**	
**Asia** *(91)*	3,161,845	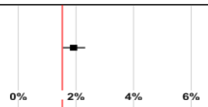	1.9% [1.6%, 2.3%]		99.84
**Europe** *(33)*	4,586,834	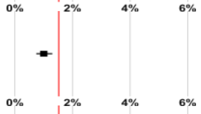	1.0% [0.7%, 1.3%]		99.87
**North America** *(19)*	33,981,458	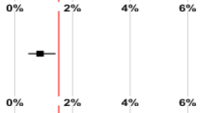	0.9% [0.5%, 1.4%]		99.99
**Oceania** *(5)*	1,096,923	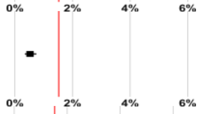	0.5% [0.3%, 0.7%]		98.16
**South America** *(2)*	15,623	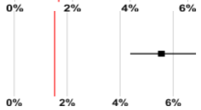	5.6% [4.4%, 6.9%]		90.30
**WHO regional classification**				**0.004**	
**WPR** *(75)*	4,030,470	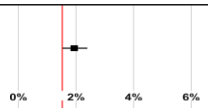	1.9% [1.5%, 2.4%]		99.90
**EUR** *(37)*	4,658,844	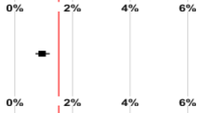	0.9% [0.7%, 1.2%]		99.85
**AMR** *(21)*	33,997,081	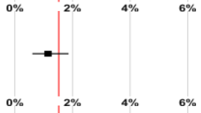	1.2% [0.6%, 1.9%]		99.99
**SEAR** *(11)*	64,585	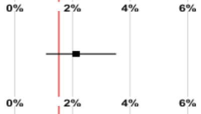	2.1% [1.1%, 3.5%]		99.15
**EMR** *(6)*	91,703	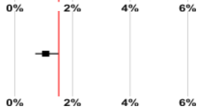	1.1% [0.7%, 1.5%]		96.91
**World Bank country classification**				**<0.001**	
**Low and Middle Income** *(89)*	3,119,749	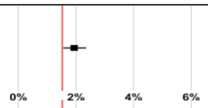	1.9% [1.6%, 2.4%]		99.85
**High Income** *(61)*	39,722,934	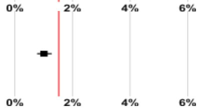	1.0% [0.8%, 1.3%]		99.98
**Country developmental status**				**<0.001**	
**Developing regions** *(94)*	3,195,938	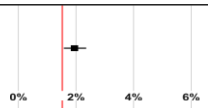	1.9% [1.6%, 2.4%]		99.84
**Developed regions** *(56)*	39,646,745	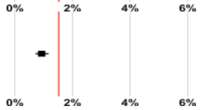	0.9% [0.7%, 1.2%]		99.98
**Peer-review status**				0.093	
**Peer-reviewed** *(143)*	40,516,932	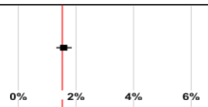	1.6% [1.3%, 1.9%]		99.97
**Not peer-reviewed** *(7)*	2,325,751	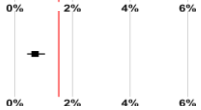	0.7% [0.4%, 1.0%]		99.81
**RoB**				0.062	

Table Sup. 1. Moderator analysis of the pooled incidence of ICP using non-outlier studies with sample sizes larger than the median sample size.

**Moderator** *(number of included effect sizes)*	**Number of pregnant women evaluated for ICP**	**Incidence [95% CI]**		**p for between-group difference**	**I^2(%)**
**Yes** *(111)*	12,099,289	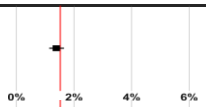	1.4% [1.1%, 1.7%]		99.93
**No** *(39)*	30,743,394	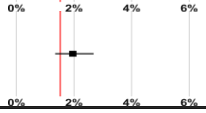	2.0% [1.3%, 2.7%]		99.99

Single effect size forest plots show the pooled effect size in that subgroup and the purple line shows the overall pooled effect size.

The cut-off was set at the median of sample sizes across the included studies.

ICP: Intrahepatic Cholestasis of Pregnancy; CI: Confidence Interval; WHO: World Health Organization; AMR: Region of the Americas; EMR: Eastern Mediterranean Region; EUR: European Region; WPR: Western Pacific Region; SEAR: South-East Asian Region; RoB: Risk of Bias.

p values faced with bold are less than the statistical significance threshold (0.05).
