# Supplementary material for: Global and regional incidence of intrahepatic cholestasis of pregnancy: a systematic review and meta-analysis
Source: BMC Med. 2025 Feb 28;23:129. doi: 10.1186/s12916-025-03935-0 (PMC11871686; doi:10.1186/s12916-025-03935-0)
Supplement: Supplementary file 6 — Additional File 6. Table of meta-regression. This table only uses non-outlier studies with larger-than-median sample sizes. [file 12916_2025_3935_MOESM6_ESM.pdf]

Table 2. Multiple univariate and multivariate meta-regression analysis.

Term	Estimate [95% CI]	p value	Estimate [95% CI]	p value
Mean maternal age	-0.01 [-0.02, 0.00]	0.072	-0.03 [-0.05, -0.02]	**<0.001**
Absolute latitude	-0.00 [-0.00, 0.00]	0.403	-0.01 [-0.01, -0.00]	**0.015**
GDM	0.05 [-0.08, 0.17]	0.438	0.36 [-0.07, 0.79]	0.102
Study start year	0.00 [-0.00, 0.00]	0.052	-0.00 [-0.01, 0.00]	0.243
RoB score	-0.01 [-0.02, 0.00]	0.183	-0.02 [-0.07, 0.02]	0.301
Multiple pregnancies	0.05 [-0.02, 0.12]	0.145	0.01 [-0.08, 0.10]	0.815
Nulliparous	0.03 [-0.06, 0.11]	0.532	0.01 [-0.13, 0.14]	0.920
Preterm labor	0.28 [0.12, 0.45]	**<0.001**		
Vaginal delivery	-0.10 [-0.19, -0.01]	**0.027**		
Undereducation	0.19 [-0.02, 0.39]	0.078		
Study midpoint	0.00 [-0.00, 0.00]	0.083		
Mean BMI	0.01 [-0.00, 0.01]	0.167		
Study end year	0.00 [-0.00, 0.00]	0.176		
Publication year	0.00 [-0.00, 0.00]	0.193		
HBS	0.10 [-0.09, 0.29]	0.313		
Smoker	-0.19 [-0.59, 0.21]	0.357		
Preeclampsia	0.25 [-0.34, 0.85]	0.405		
Mean parity	0.01 [-0.02, 0.05]	0.408		
HTN	-0.22 [-0.77, 0.32]	0.420		
Stillbirth	2.02 [-3.12, 7.16]	0.441		
GHTN	0.12 [-0.58, 0.82]	0.741		
PROM	0.03 [-0.25, 0.30]	0.845		
ART	-0.00 [-0.06, 0.06]	0.955		
Hepatitis C	0.07 [-3.09, 3.23]	0.965		
DM	-0.01 [-1.36, 1.34]	0.991		

Multiple independent univariate regression models were produced. Brute-force approach was used to model all possible combinations of ten moderators with the least shared missing values. Then, the best model was chosen using AICc.
p values faced with bold are less than the statistical significance threshold (0.05).
AICc: corrected Akaike Information Criterion; CI: Confidence Interval; GHTN: Gestational Hypertension; GDM: Gestational Diabetes Mellitus; PROM: Premature Rupture of Membrane; DM: Diabetes Mellitus; HTN: Hypertension; BMI: Body Mass Index; ART: Assisted Reproductive Technology.
A similar table presenting results of meta-regression when outlier studies and those that have sample sizes less than median were excluded is available in Additional File 10.
